# Prediction of essential oil content in spearmint (*Mentha spicata*) via near-infrared hyperspectral imaging and chemometrics

**DOI:** 10.1038/s41598-023-31517-8

**Published:** 2023-03-14

**Authors:** Sam Van Haute, Amin Nikkhah, Derick Malavi, Sajad Kiani

**Affiliations:** 1 Department of Food Technology, Safety and Health, Faculty of Bioscience Engineering, Ghent University, Coupure Links 653, 9000 Ghent, Belgium; 2 Department of Molecular Biotechnology, Environmental Technology, and Food Technology, Ghent University Global Campus, 119, Songdomunhwa-Ro, Yeonsu-Gu, Incheon, 21985 South Korea; 3 Friedman School of Nutrition Science and Policy, Tufts University, Boston, MA USA; 4 Biosystems Engineering Department, Sari Agricultural Sciences and Natural Resources University, Sari, Iran

**Keywords:** Computational biology and bioinformatics, Plant sciences

## Abstract

Spearmint (*Mentha spicata* L.) is grown for its essential oil (EO), which find use in food, beverage, fragrance and other industries. The current study explores the ability of near infrared hyperspectral imaging (HSI) (935 to 1720 nm) to predict, in a rapid, nondestructive manner, the essential oil content of dried spearmint (0.2 to 2.6% EO). Spectral values of spearmint samples varied considerably with spatial coordinates, and so the use of averaging the spectral values of a surface scan was warranted. Data preprocessing was done with Multiplicative Scatter Correction (MSC) or Standard Normal Variate (SNV). Selection of spectral input variables was done with Least Absolute Shrinkage and Selection Operator (LASSO), Principal Component Analysis (PCA) or Partial Least Squares (PLS). Regression was executed with linear regression (LASSO, PLS regression, PCA regression), Support Vector Machine (SVM) regression, and Multilayer Perceptron (MLP). The best prediction of EO concentration was achieved with the combination of MSC or SNV preprocessing, PLS dimension reduction, and MLP regression (1 hidden layer with 6 nodes), achieving a good prediction with a ratio of performance to deviation (RPD) of 2.84 ± 0.07, an R^2^ of prediction of 0.863 ± 0.008, and a RMSE of prediction of 0.219 ± 0.005% EO. These results show that NIR-HSI is a viable method for rapid, nondestructive analysis of EO concentration. Future work should explore the use of NIR in the visible spectrum, the use of HSI for determining EO in other plant materials and the potential of HSI to determine individual compounds in these solid plant/food matrices.

## Introduction

Spearmint (*Mentha spicata* L.) is a plant species belonging to the Lamiaceae family. The genus *Mentha* contains a number of commercially grown species, such as corn/Japanese mint (*Mentha arvensis*), peppermint (*Mentha piperita*), bergamot mint (*Mentha citrate*), and spearmint (*Mentha spicata*)^1–3^. Spearmint is cultivated for its essential oils (EOs), which are used in several industries, including the fragrances, food and beverage industries, and for its health-beneficial properties (antioxidant, anti-inflammatory and antimicrobial)^3–5^. In food applications such as in chewing gum and confectioneries the extracted EOs are used. In traditional foods, the leaves (fresh or dried) are added as a flavouring agent in e.g. soup, bread, salad, cheese and herbal teas^1,6^. EOs are liquid extractions of aromatic plants that consist of volatile compounds and which are commonly acquired through steam distillation^7^. The major compounds in the spearmint EOs are the monoterpenoid carvone and the monoterpene limonene^8,9^. Besides the sensorial applications, Spearmint EO also shows antimicrobial and antioxidant effects^6,8^ and is being experimented with as natural antimicrobial nonthermal treatment for animal and vegetable foods^10–12^.

The qualitative characteristics of spearmint can vary, including variations in EO quantity, as well as the molecular composition of the EO. Certain traits of *M. spicata* plants such as main stem length and dry weight can indicate larger EO yields, which can be helpful in breeding practices with the goal of EO yields increase^13^. Gaining a rapid estimate of the EO content of a particular herb, without the need for destroying the herb in the measuring process, is valuable information. Hyperspectral imaging (HSI) is a rapid and nondestrutive technology, with the potential of gaining chemical information of imaged objects. The strength of HSI is the lack of sample preparation, absence of sample destruction, rapid analysis, and the possibility to gain both spatial and spectral information. An HSI image differs from a Red–Green–Blue image in that every pixel contains a more extensive spectrum, for example a near infrared (NIR) spectrum, as is the case in this study^14^.

Different chemometric methods were explored in this study in order to extract the relevant spectral information and apply it in models for predicting the EO concentrations. Using all NIR-HSI spectral variables in multilinear regression (MLR) is not an option due to the high collinearity among spectral variables^15^. In order to overcome the low performance of MLR and to reduce the chance that spectral noise becomes part of the model architecture (leading to overfitting), the use of (i) statistical techniques based on latent variables (LVs), i.e. Principal Component Regression (PCR) and PLS, (ii) the use of Least Absolute Shrinkage and Selection Operator (LASSO) regression to reduce the number of input variables (NIR wavelengths), and (iii) the machine learning tools Support Vector Machine (SVM) and multilayer perceptron (MLP) were applied in this study.

There are hardly any studies that deal with using hyperspectral imaging (or NIR) to detect total EO content in herb and plant samples. One study, by^16^, determined the amount of EOs (obtained by steam distillation) in Sichuan pepper (*Zanthoxylum bungeanum* Maxim.) with HSI in the range 380–1040 nm. Nonetheless, the possibility of using NIR spectral information to predict components that occur in (or are related to components that occur in) spearmint EO has been proven through NIR spectroscopy studies on related compounds. NIR spectroscopy was used to quantify the monoterpene citral in spray dried, dextrin/lecithin encapsulated microparticles^17^. In another study, Beć et al.^18^ used NIR spectroscopy to quantify limonene (major compound in spearmint EO) in citrus oil. Considering the “in principle” suitability of NIR to provide nondestrutive information about EO compounds similar to those occurring in spearmint EO, the current study used NIR-HSI hyperspectral imaging to assess a solid matrix, in combination with chemometric techniques, to inquire nondestructively about the quantity of total EOs in spearmint dried leaves, which has not been attempted before.

## Materials and methods

### Collection of samples

**Figure 1:**
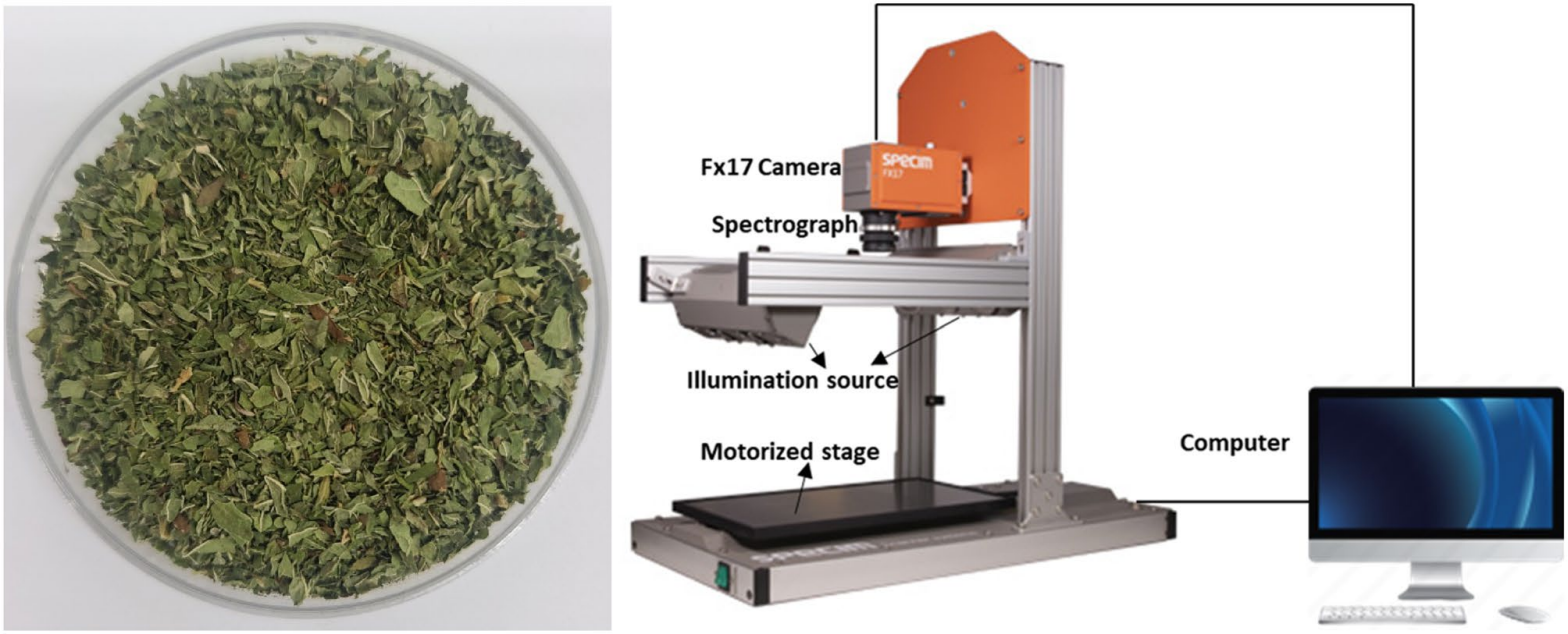
(**A**) Image of a dried spearmint sample. (**B**) Setup of the hyperspectral camera.

Fifty-eight samples of spearmint were collected from different geographic locations in Iran (Table 1). The collection of plant material complied with relevant institutional, national, and international guidelines and legislation. The aerial parts were harvested at onset of flowering and subsequently air dried at room temperature until constant weight (25 ± 1 °C) was reached (Fig. 1A).

**Table 1:** Description of origin of spearmint samples and EO concentrations.

	Central	East	North	South	West
City of sampling	Anbar shahrood	Ardabil	Farangi jiroft	Bookan	Bandarabas
	Barzak kashan	Ghaemshahr	Iranshahr	Boroojerd	Booshehr
	Esfahan	Noshahr	Jiroft	Dezful	
	Ghazvin		Neishaboor	Ghorve	
	Majarestan		Sabzevar	Marivan	
	Semnan			Shooshtar	
	Shahrood				
	Shiraz				
	Tarbiat modares				
	Varamin				
	Vordavord				
Sample size	20	5	13	14	6
Minimum–maximum	0.38–2.6	0.25–1.43	0.4–2.25	0.2–2.23	0.35–2.45
Mean + standard deviation	1.04 ± 0.55	1.00 ± 0.47	1.00 ± 0.52	1.00 ± 0.71	1.22 ± 0.77

### Extraction procedure and quantification of EO

EO was extracted from the mint samples according to^13^ through hydrodistillation. Fifty g of dried sample and 500 mL of distilled water were added to a flask and subjected to Clevenger-type apparatus processing for 3 h to isolate the EO. EO samples were collected in glass vials, dried with anhydrous sodium sulfate, and stored at 4 °C for further processing. The EO content was calculated based on dry weight of the mint samples.

### HSI data collection of mint samples

Images were acquired from dried mint samples using near-infrared (935–1720 nm) hyperspectral imaging (Fx17e Specim, Spectral Imaging Oy Ltd, Finland). The weight for all the samples was standardized to 10 g before the acquisition of images. The HSI system comprised of the following: an Fx17 hyperspectral camera fitted with a front lens, an imaging spectrograph and an image sensor, halogen-based illumination consisting of six tungsten lamps, a displacement system (40 × 20 Specim Lab Scanner), and a computer (Fig. 1B). Acquisition of images was controlled from the computer via the Lumo scanner software. The optimal exposure time, frame rate, and platform speed parameters for acquiring the images were 7.00 ms, 19.50 Hz, and 2.6 mm/s, respectively. The sample was scanned in the 935–1720 nm spectral range with a spectral width of 3.5 nm. Each hyperspectral image was a hypercube with 672 × 512 pixels (x and y dimension) and 224 bands (λ/z dimension).

Some variation exists in the surface of the sample because of how the individual dried mint pieces are scattered and oriented (Fig. 1A), and which would influence the scattering and reflection of the NIR radiation. This can be solved in part by taking a large enough region of interest of the image (50 × 50 pixels) and averaging these pixels. In addition, a setup was used in which from each mint sample 3 subsamples were created to account for differences in the orientation of the dried mint leaf pieces, which would influence the scattering and reflection of the NIR radiation. Each subsample was recorded 3 times, and the resulting images were averaged. This was done for each of the 3 subsamples, resulting in 58 × 3 = 174 imaged samples that were introduced as the spectral data during the chemometrics part.

### Image correction

Image correction and normalization were performed by classic ENVI (IDL 8.7.2) software. The raw image was first calibrated using the black and white reference images according to Eq. (1):

1 $${\text{R}} = \left( {{\text{I}} - {\text{B}}} \right)/\left( {{\text{W}} - {\text{B}}} \right)$$

where R is the corrected hyperspectral image, I is the raw hyperspectral image of the sample, W is the white reference image of a standard white calibration board (99.9% reflectance), and B is the dark image (0%) reflectance acquired by automatically closing the shutter. The corrected image was then normalized by scaling the range of pixel intensity values to between 0 and 1 (reflectance).

A 50 × 50 pixels region of interest was selected from the processed image at the center of the sample to extract the average spectral reflectance of the sample. Extraction of ROI was executed in IDL ENVI (version 5.5.2) software.

### Data preprocessing

Prior to construction of models, HSI spectra were subjected to pre-processing. Spectral pre-processing enhances the quality of spectral data and reduces information from undesirable effects such as light scattering, particle-size effects, and morphological differences^19^. Standard Normal Variate (SNV) and Multiplicative Scatter Correction (MSC) were investigated in this study. SNV and MSC are capable of removing additive and multiplicative light scattering effects from non-uniform sample surfaces such as mint samples in our study^20^. HSI spectra pre-treatment was performed by Unscrambler X, CAMO Software AS (version 10.4, Oslo, Norway).

### Modelling

**Figure 2:**
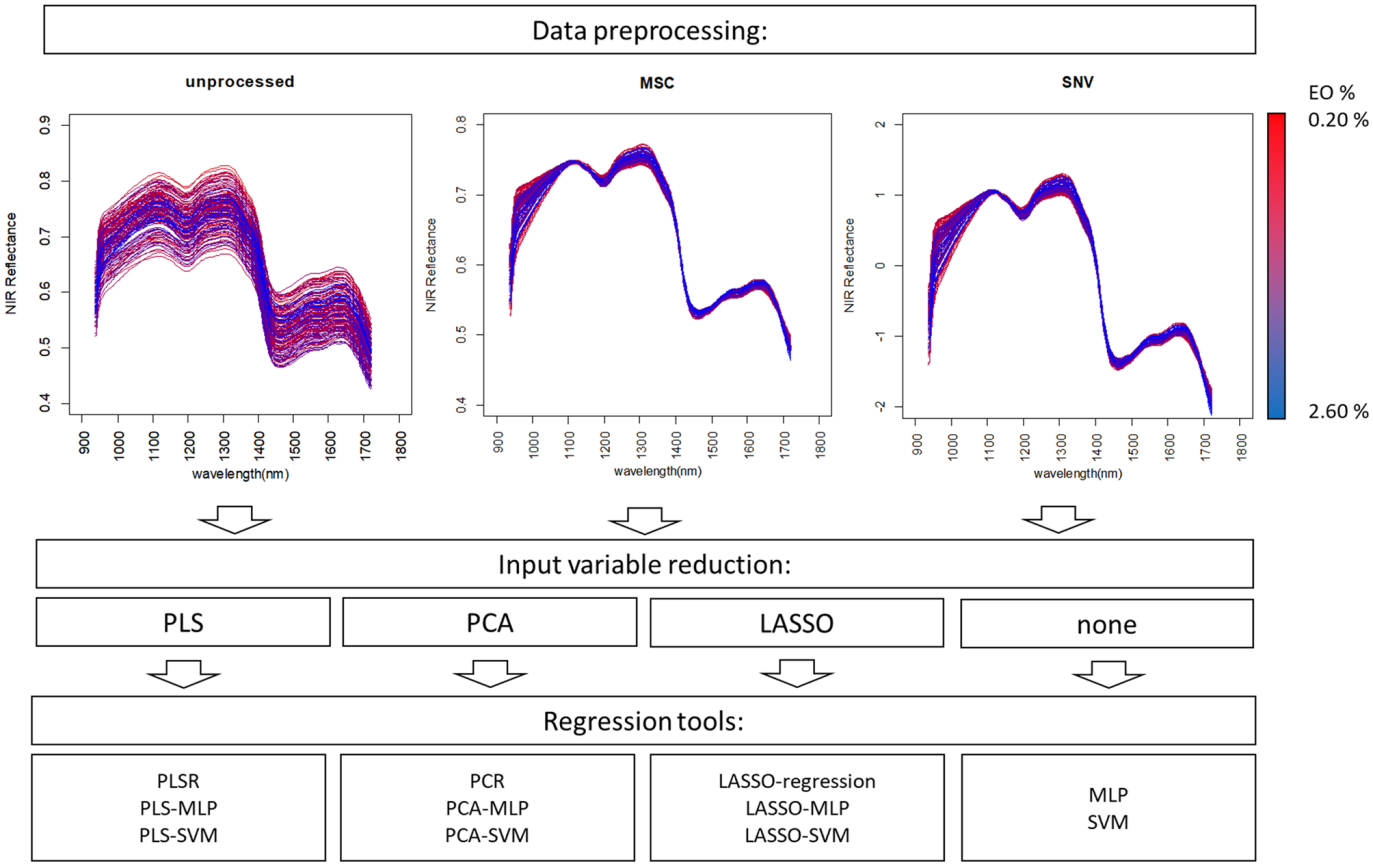
Overview of preprocessing, variable selection and regression tools used to predict the EO concentration in spearmint.

**Figure 3:**
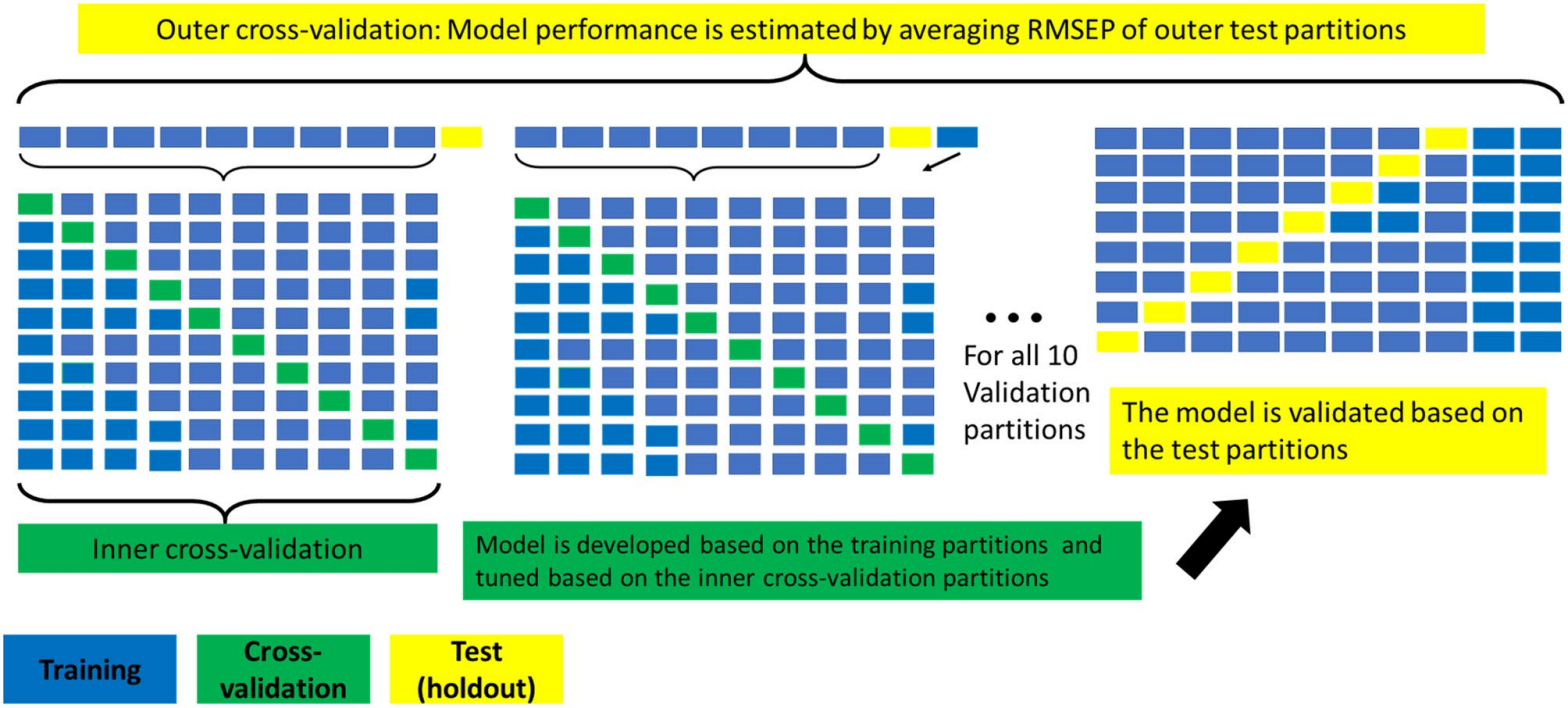
Overview of nested k-fold cross-validation. Model construction is done via training data. Model optimization was done via inner cross-validation. Model performance was tested via prediction on the test data.

An overview of the data preprocessing, input variable selection, and used regression tools is shown in Fig. 2. For every regression tool, some parameter(s) needed optimization. Nested tenfold cross-validation was used to assess the performance of the models (Fig. 3). At the start, a portion of data (10% of the samples) is split off for use as testing data (holdout set). The rest of the data (90% of the samples) is used for constructing the model, including feature selection and parameter tuning, based on cross-validation (the 90% data is divided into tenfolds). The test set is then used to validate the model. This is repeated by each time splitting off another 10% of the data to be used as testing data, and constructing and tuning the model, until all the data is used once for testing. In this manner, the test data of a certain iteration of outer cross-validation is not used to optimize the performance of the model, providing a more reliable way for choosing the optimal model than regular cross-validation. In cases where the data set is not very large, nested cross-validation can produce robust and unbiased performance estimates, and can be an economical alternative if testing of the models with a separate dataset is not feasible due to limited size of the dataset^21,22^. Optimization was reached when the minimum Root Mean Squared Error of Cross-validation RMSECV was determined and tested by determining the RMSE of prediction (RMSEP) of the validation (test sets). To improve the estimate of the prediction error, the model at optimal settings was validated with 10 times repeated nested tenfold cross-validation. By comparing the RMSECV and RMSEP (error of holdout testing), it was possible to better detect the presence of overfitting in the different models. Overfitting of a model means that the model contains spectral information that does not contribute to predicting an aspect of the total population of the target object (e.g. EO concentration of mint samples) but only to predicting the subset of samples used to build/train the model^23,24^.

PCR is a well-known technique where PCA is first applied to reduce the spectral variables to a set of principal components or (latent variables), followed by MLR on (a subset of) the principal components. The principal components or uncorrelated, which solves the issue of collinearity of MLR^25^. PLS regression constructs latent variables in such a way that they are oriented along directions of maximal covariance between spectral variables and the response variable. This ensures that latent variables are ranked according to contribution to the prediction quality of the regression model, making it easier to select for a parsimonious model without overfitting than in the case of PCR^23,24^. LASSO regression aims to select the input variables that lead to minimizing the prediction error of a regression model and discard the other input variables. This is done by imposing a constraint on the variable coefficients, by shrinking the coefficients towards zero, forcing the sum of the absolute value of the coefficients to be below a chosen value (denoted as lambda λ). As such, some of the variables end up with a zero value coefficient and the number of input variables is ultimately reduced. As such, LASSO-regression not only serves for prediction purposes but can also be used for spectral input variable selection (feature selection) for other regression tools, such as machine learning tools^26^.

Furthermore, machine learning tools were used to solve the regression problem, namely SVM and MLP. SVM was originally developed to solve classification problems^27^, but it is also applicable for regression purposes, including spectral chemometrics. For a general overview of the fundamentals of SVM, the original work by^27^ is recommended as well as a comprehensive explanation by^28^. For parameter estimation of SVM regression models, the authors refer to^29^. In the SVM models, a kernel function is determined which can be: linear, polynomial, radial basis or sigmoid. For the regression models in the spearmint dataset, the linear kernel function always generated superior prediction results. Initial parameter values were chosen based on the work of^29^. Parameters were further adjusted based on primary grid searching. In order to find optimal settings for the SVM models, the epsilon Ɛ parameter (0.9,0.7,0.5,0.3,0.2,0.1,0.05,0.02,0.01,0.005,0.001) and the “cost of constraints violation” C parameter (0.01,0.1,0.5,0.75,1,3,5,7,10,25, followed by jumps of 25 till 300) were varied to perform a grid search. The Ɛ represents the ‘tolerance margin” where no penalty is given if training cases in the regression do not deviate more from the hyperplane (basically the best fit line for prediction) than the allowed Ɛ. If this value is high, a high error is allowed and potentially certain data trends are not considered in the model (underfitting). On the other hand, if this value is low, the allowed error is lower but this increases the chance of overfitting. The C parameter controls the penalty that is imposed on cases which are outside of the regression tolerance margin (which was set based on the Ɛ). If C is large, then cases outside of the tolerance margin are heavily penalized, decreasing the training bias, but increasing the variance in prediction and as such potentially leading to overfitting, whereas low values of C can lead to a higher training bias^28,29^.

MLP is a type of fully connected, feedforward artificial neural network, which applies neurons (software nodes) in layers, and connects inputs with outputs through these layer(s) of neurons. In its simplest form (and as it is applied in this study) an MLP contains an input layer which takes in the input, i.e. hyperspectral reflectance variables (or another set of inputs acquired from the variable selection process), a hidden layer of neurons which are connected to the input layer, and an output layer which connects to the output variable, i.e. the EO concentration^30^. For the hidden and output layers the hyperbolic tangent activation function (“tansig”) and simple identity activation function (“linear”) were used, respectively. In order to train the network, a number of training cycles (epochs) was done for each model architecture, i.e., the number of neurons in the hidden layer. If too much training is done, the model will suffer from overfitting and fail at accurately predicting the testing data. In the initial grid search, the number of neurons was varied from 1 to 10 and the number of training epochs from 1, followed by a search in the interval between 5 and 200 training epochs in steps of 5 epochs.

For both SVM and MLP models, additional searching could be done after the initial grid search in the region with lowest RMSECV. When PCA and PLS were used as dimension reduction tools for SVM/MLP, an additional parameter, i.e., the number of PCs/LVs was added to the grid search. The search for PCs was done in increments of 5 PCs, the search for LVs in increments of 2. When the region with lowest RMSECV was detected, a more detailed search was done where the PCs/LVs were increased by 1 at the time. More info on how LASSO, PCA and PLS were coupled to SVM and MLP is provided in “supplementary information” and Fig. S1.

### Model performance assessment

The optimalization of the model settings was evaluated by calculating the RMSECV and the Residual Predictive Deviation (RPD). RPD is the ratio of the standard deviation of the measured dependent variable (adulterant’s concentration) values to the RMSECV. RPD values lower than 2.0 are considered insufficient for prediction while values between 2.0 and 2.5 are sufficient for approximate quantitative predictions. Higher values are indicators of good (between 2.5 and 3.0) and excellent (> 3) predictions^31,32^. When the optimal model settings were determined, the RMSEP and RPD of prediction (RPDp) were further used as holdout set validation.

### Comparison of NIR point measurements with NIR surface scanning

In this study, models are based on collecting the NIR spectrum at many different spatial locations of the sample (scan of 50 × 50 pixels). In order to assess whether this amount of information collection is necessary, a “point” (data from 1 pixel of the image) collection approach was executed as a comparison. At 30 random spatial locations of a sample, the NIR spectrum of 1 pixel was collected with IDL ENVI (version 5.5.2). This was done for 3 samples, 1 with a low EO concentration (0.25%), 1 with an average EO concentration (1.33%), and 1 with a high EO concentration (2.45%). The best model settings (acquired from the protocol as described in 2.6 and 2.7) were used to construct the model by training on all samples, except for these 3 selected ones. Afterwards, the EO concentration was predicted for the scanned samples (50 × 50 pixels) and for the point versions of the same samples (30 random point measurements). To take 30 point measurements of 1 sample is unrealistically high, yet this number was chosen in order to have sufficient data. Histograms were made to see the distribution of predicted EO of the point measurements. The Wilcoxon signed rank test (executed in SPSS Statistics 26 (IBM)) was used to compare the results with the measured EO concentrations.

### Software for modelling and statistics

Rstudio (version 1.4.1106) was used for modeling the data. The partitions for nested tenfold cross-validation were done with ‘createMultiFolds’ in the ‘hsdar’ package^33^. PLS regression models were implemented via the package ‘pls’^34^. MLP models were implemented via the package ‘monmlp’ (Cannon, 2017). SVM models were implemented via the package ‘e1071’^35^. Multilinear models, PCA and PCR were implemented via the ‘stats’ package (R Core Team, 2022). LASSO-regression was done via the “glmnet” package^36^. Counting of number of pixels with a certain color in the images was done via the package “countcolors”^37^.

Statistical comparison of the model factors (preprocessing, variable selection, regression tool) was done in SPSS statistics 26 by General Linear Model (GLM) analysis, of the form RPD = f(preprocessing, variable selection, regression tool) to assess the significance of these factors and post-hoc analysis was done with Tukey HSD (p < 0.05).

## Results and discussion

### Mint samples

The aerial parts of 58 spearmint samples from different regions in Iran were collected (Table 1). Ranges of EO concentrations were very similar among regions (ANOVA, p = 0.95), so geography did not seem to have an impact on the EO quantities. The EO concentration in the current samples was between 0.20 and 2.60% (g/100 g dry matter). An earlier study analyzed spearmint samples from the island of Crete (Greece) where EO concentrations between 1.2 and 3.9% (g/100 g dry matter) were measured^38^. Another study in the Molise Region in Italy reported spearmint EO concentrations of 0.2 to 1.3% (g/100 g dry matter)^8^.

### Prediction of essential oil concentration in spearmint samples

The choice of regression tool was of great significance for EO prediction quality (GLM, p = 10^–15^), with MLP > SVM ≈ multilinear models (based on Tukey tests). The superior prediction performance of MLP can potentially be attributed in part to the ability to deal with the spectral data in a nonlinear fashion, whereas PLS and other linear regression techniques cannot^39^. Multilinear regression (PCR, PLS, LASSO-regression) was not very efficient (RPDp between 2.20 and 2.45) at making EO predictions (Table 2).

**Table 2:** Performance of EO concentration prediction models.

Regression toolΩ	Preprocessing	Variable selection	Details	R^2^cv*	RMSECV	RPD	R^2^p	RMSEP	RPDp	RPDp groups ΩΩΩ (Tukey post-hoc)
Multilinear (LASSO regression)	None	LASSO	λ = 1.6 × 10^–4^	0.771 ± 0.001**	0.279 ± 0.001	2.10 ± 0.01	0.795 ± 0.006	0.274 ± 0.004	2.20 ± 0.04	A******
Support vector machine	None	PCA	Ɛ = 0.6,cost = 0.25, 30 PCs,kernel = linear,SVs = 32ΩΩ	0.777 ± 0.001	0.276 ± 0.001	2.12 ± 0.01	0.798 ± 0.006	0.268 ± 0.003	2.23 ± 0.03	A******
Support vector machine	None	None	Ɛ = 0.48,cost = 110,kernel = linear,SVs = 57	0.787 ± 0.001	0.270 ± 0.001	2.17 ± 0.01	0.803 ± 0.006	0.266 ± 0.004	2.26 ± 0.04	AB*****
Support vector machine	None	LASSO	Ɛ = 0.5,cost = 100,kernel = linear,SV = 53	0.788 ± 0.005	0.271 ± 0.006	2.15 ± 0.03	0.820 ± 0.013	0.259 ± 0.010	2.26 ± 0.06	AB*****
Multilayer perceptron	None	LASSO	Nodes = 1, iterations = 170	0.781 ± 0.003	0.275 ± 0.002	2.11 ± 0.01	0.800 ± 0.007	0.259 ± 0.002	2.27 ± 0.03	ABC****
Multilinear (PCA regression)	None	PCA	PCs = 32	0.790 ± 0.001	0.268 ± 0.001	2.18 ± 0.01	0.808 ± 0.006	0.265 ± 0.004	2.27 ± 0.04	ABC****
Multilinear (PCA regression)	MSC	PCA	PCs = 25	0.789 ± 0.001	0.268 ± 0.001	2.18 ± 0.01	0.803 ± 0.007	0.266 ± 0.004	2.27 ± 0.04	ABC****
Multilinear (PLS regression)	None	PLS	LVs = 15	0.793 ± 0.001	0.267 ± 0.001	2.19 ± 0.01	0.809 ± 0.006	0.265 ± 0.004	2.27 ± 0.04	ABC****
Multilayer perceptron	None	PCA	Nodes = 1,iterations = 150,PCs = 30	0.792 ± 0.006	0.268 ± 0.001	2.19 ± 0.01	0.801 ± 0.008	0.268 ± 0.005	2.27 ± 0.04	ABC****
Support vector machine	SNV	LASSO	Ɛ = 0.5,cost = 10,kernel = linear,SVs = 44	0.802 ± 0.005	0.262 ± 0.005	2.22 ± 0.03	0.820 ± 0.014	0.255 ± 0.01	2.31 ± 0.10	ABCD***
Multilinear (LASSO regression)	MSC	LASSO	λ = 5.0 × 10^–4^	0.789 ± 0.001	0.269 ± 0.001	2.18 ± 0.01	0.808 ± 0.001	0.261 ± 0.004	2.33 ± 0.05	ABCDE**
Multilinear (LASSO regression)	SNV	LASSO	λ = 5.0 × 10^–4^	0.788 ± 0.001	0.270 ± 0.001	2.17 ± 0.01	0.809 ± 0.007	0.261 ± 0.004	2.34 ± 0.05	ABCDE**
Support vector machine	MSC	LASSO	Ɛ = 0.5,cost = 25,kernel = linear, SVs = 42	0.802 ± 0.006	0.263 ± 0.005	2.21 ± 0.04	0.829 ± 0.012	0.253 ± 0.012	2.34 ± 0.10	ABCDE**
Multilayer perceptron	None	None	Nodes = 3, iterations = 150	0.762 ± 0.006	0.292 ± 0.006	2.03 ± 0.02	0.809 ± 0.008	0.266 ± 0.008	2.34 ± 0.06	ABCDE**
Support vector machine	None	PLS	Ɛ = 0.65,cost = 100,LVs = 18,kernel = linear,SVs = 30	0.796 ± 0.001	0.265 ± 0.001	2.21 ± 0.01	0.814 ± 0.006	0.257 ± 0.004	2.35 ± 0.04	ABCDE**
Multilinear (PCA regression)	SNV	PCA	PCs = 35	0.796 ± 0.001	0.265 ± 0.001	2.21 ± 0.01	0.816 ± 0.006	0.258 ± 0.004	2.36 ± 0.05	ABCDE**
Support vector machine	MSC	PCA	Ɛ = 0.5,cost = 0.1,PCs = 30,kernel = linear, SVs = 55	0.805 ± 0.001	0.258 ± 0.001	2.26 ± 0.01	0.819 ± 0.006	0.254 ± 0.004	2.37 ± 0.04	ABCDE**
Support vector machine	SNV	PCA	Ɛ = 0.5,cost = 0.1,PCs = 29,kernel = linear,SVs = 53	0.801 ± 0.001	0.261 ± 0.001	2.24 ± 0.01	0.818 ± 0.006	0.254 ± 0.004	2.38 ± 0.04	ABCDE**
Support vector machine	SNV	None	Ɛ = 0.5,cost = 3,kernel = linear,SVs = 53	0.805 ± 0.001	0.258 ± 0.001	2.27 ± 0.01	0.819 ± 0.006	0.253 ± 0.004	2.39 ± 0.04	ABCDE**
Support vector machine	MSC	None	Ɛ = 0.5,cost = 3,kernel = linear, SVs = 57	0.806 ± 0.001	0.257 ± 0.001	2.27 ± 0.01	0.820 ± 0.006	0.252 ± 0.004	2.40 ± 0.04	ABCDE**
Multilinear (PLS regression)	MSC	PLS	LVs = 13	0.815 ± 0.001	0.252 ± 0.001	2.32 ± 0.01	0.827 ± 0.006	0.249 ± 0.004	2.44 ± 0.05	ABCDEF*
Multilinear (PLS regression)	SNV	PLS	LVs = 14	0.814 ± 0.001	0.252 ± 0.001	2.32 ± 0.01	0.826 ± 0.006	0.249 ± 0.004	2.45 ± 0.05	ABCDEF*
Support vector machine	MSC	PLS	Ɛ = 0.65,cost = 1,LVs = 14,kernel = linear,SVs = 11	0.814 ± 0.001	0.252 ± 0.001	2.33 ± 0.01	0.826 ± 0.006	0.247 ± 0.004	2.45 ± 0.04	ABCDEF*
Multilayer perceptron	SNV	LASSO	Nodes = 9, iterations = 80	0.822 ± 0.003	0.252 ± 0.003	2.32 ± 0.02	0.812 ± 0.014	0.252 ± 0.006	2.45 ± 0.06	ABCDEF*
Support vector machine	SNV	PLS	Ɛ = 0.5,cost = 0.5,LVs = 16,kernel = linear,SVs = 34	0.816 ± 0.001	0.250 ± 0.001	2.34 ± 0.01	0.828 ± 0.006	0.246 ± 0.004	2.48 ± 0.05	ABCDEFG
Multilayer perceptron	None	PLS	LVs = 15, nodes = 3, iterations = 60	0.827 ± 0.001	0.244 ± 0.001	2.40 ± 0.01	0.835 ± 0.006	0.243 ± 0.004	2.50 ± 0.05	ABCDEFG
Multilayer perceptron	SNV	PCA	Nodes = 11,iterations = 25,PCs = 25	0.824 ± 0.002	0.248 ± 0.002	2.37 ± 0.05	0.841 ± 0.007	0.237 ± 0.005	2.59 ± 0.06	*BCDEFG
Multilayer perceptron	MSC	LASSO	Nodes = 9,iterations = 90	0.834 ± 0.002	0.243 ± 0.002	2.40 ± 0.02	0.844 ± 0.008	0.235 ± 0.006	2.61 ± 0.06	**CDEFG
Multilayer perceptron	MSC	PCA	Nodes = 11, iterations = 25,PCs = 25	0.827 ± 0.002	0.247 ± 0.001	2.38 ± 0.01	0.836 ± 0.008	0.239 ± 0.005	2.62 ± 0.07	**CDEFG
Multilayer perceptron	SNV	None	Nodes = 8,iterations = 80	0.813 ± 0.003	0.259 ± 0.005	2.27 ± 0.02	0.844 ± 0.008	0.238. ± 0.006	2.65 ± 0.08	***DEFG
Multilayer perceptron	MSC	None	Nodes = 9,iterations = 60	0.833 ± 0.002	0.243 ± 0.002	2.42 ± 0.02	0.847 ± 0.008	0.234 ± 0.006	2.66 ± 0.06	****EFG
Multilayer perceptron	SNV	PLS	LVs = 14, nodes = 6, iterations = 100	0.839 ± 0.001	0.237 ± 0.001	2.48 ± 0.01	0.866 ± 0.006	0.218 ± 0.012	2.83 ± 0.07	******G
Multilayer perceptron	MSC	PLS	LVs = 13, nodes = 6, iterations = 60	0.844 ± 0.002	0.232 ± 0.001	2.53 ± 0.01	0.863 ± 0.008	0.219 ± 0.005	2.84 ± 0.07	******G

Ω ranking of models in this table is done according to increasing RPD, ΩΩ SVs = number of support vectors, ΩΩΩ Different letters denote significant difference according to Tukey post-hoc test,* R^2^cv means R^2^ of cross-validation, R^2^p means R^2^ of holdout validation, ** standard error.

LASSO-regression can itself perform regression with wavelength selection, but it is not quite competitive with some other multivariate regression tools, especially when the number of samples is lower than the number of input variables as in many studies that deal with spectral datasets^40^. Performances of SVM and PLS multilinear regression models were not significantly different in this study. On the other hand, Ke et al.^16^ observed that, for determination of EO in Sichuan pepper, PLS regression performed less effective than SVM regression and Extreme Learning Machine (which is a type of feedforward neural network without tuning of the weights of the hidden nodes).

Variable selection was also of significance in the prediction of EO % (GLM, p = 10^–6^), with PLS > PCA≈ “no variable selection” > LASSO. PLS was significantly better as a tool to reduce the spectral variables for subsequent use by the regression tools than were the other methods. Interestingly, LASSO actually resulted in a worse selection of spectral variables than using the entire set of spectral variables for the regression tools SVM and MLP. Again, this can be explained by this type of spectral dataset in which the number of spectral inputs is larger than the number of cases^40^. This becomes clear when observing which variables were selected by the LASSO algorithm. When LASSO is applied on the unprocessed spectra, most variables are selected for the regression (Fig. S2A). For the MSC and SNV (Fig. S2B and C) the number of selected spectral variables was greatly reduced, but still variation could be seen in the percentage of inclusion in the LASSO trials (being 100 trials from nested tenfold cross-validation). Basically, the choice of spectral variables depended on the composition of the training set and as such overfitting happened during training and the RMSECV increased because of it.

Preprocessing had a significant influence on the model prediction accuracy (GLM, p = 10^–12^) as well, with MSC ≈SNV > “unprocessed” spectra. The 8 best models were all constructed with MLP and of these the 7 models with the highest RPDp (between 2.50 and 2.84) used SNV or MSC as preprocessing (Table 2). Interestingly, MLP was good at predicting the EO concentration, even without variable selection, as long as preprocessing was done, with RPDp of 2.65 after SNV preprocessing and 2.66 after MSC preprocessing (Table 2). However, when no spectrum preprocessing was done, MLP was only decent at predicting the EO % after PLS variable selection (RPDp 2.50), whereas the other MLP models without preprocessing had lower prediction efficiencies (RPDp between 2.27and 2.34). This illustrates the importance of preprocessing of spectral data before application as input variables. In most studies on hyperspectral imaging and MLP, variable selection techniques are included to some degree. However, Vásquez et al.^39^ predicted Swiss-type cheese ripening with HSI (range 400 to 1000 nm) with MLP as regression tool and this with both the full set of spectral input variables, as well as a selection of input variables (based on PLS loadings), and better prediction was observed with the full set of spectral variables.

**Figure 4:**
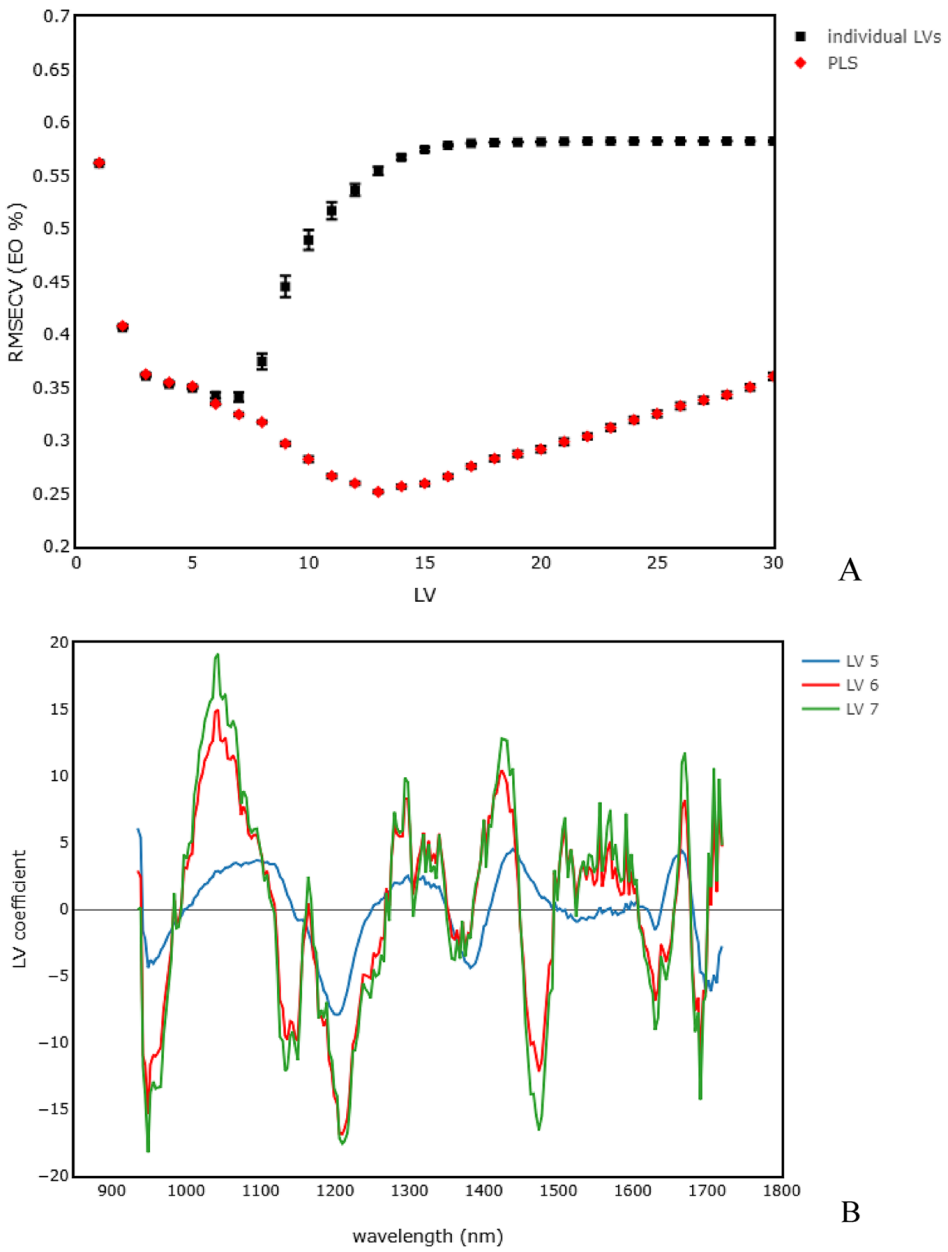
(**A**) RMSE of the linear regression between EO % and the predicted values of each of the individual PLS LVs or linear combination of LVs (i.e. PLS) of MSC preprocessed spectral data; (**B**) LV coefficients of LVs 5, 6, 7 of MSC preprocessed data.

The best models in this study were achieved with MSC or SNV preprocessing, PLS variable selection and MLP regression (Table 2) with the MLP PLS MSC having a slightly higher RPD (2.53 ± 0.01) than the MLP PLS SNV model (2.48 ± 0.01), whereas the RPDp of both models was virtually the same with RPDp of 2.83 ± 0.07 for MLP PLS SNV and RPDp of 2.84 ± 0.07 for MLP PLS MSC. Taking a closer look at these models, with the MLP PLS MSC as example, the relation between the individual PLS LVs of MSC preprocessed data and the measured EO %, LVs 5,6 and 7 had the lowest RMSECV values (Fig. 4A), and therefore provided the best fit between the spectral variables and the EO % values. By inspecting the coefficients of LVs 5 to 7, some indicative information related to the relative importance of the spectral variables could be obtained (Fig. 4B). Absorption of NIR is due to overtones and combination tones of vibrations involving C–H, O–H, and N–H chemical bonds present in compounds such as proteins, carbohydrates, water, polyphenols, alkaloids, aroma compounds, volatile and nonvolatile acids^41,42^. Dominant bands were observed in regions around 1200–1213 nm (C–H second overtone of –CH3–, –CH=CH–, and –CH2– groups), 1386 (a –CH2 structure), 1400–1450 nm (potentially attributed to aliphatic alcohols, and phenols and carbonyl groups, e.g., ketones and aldehydes, O–H polymeric groups from complex carbohydrates and O–H stretching of water), 1474 (N–H stretch first overtone and O–H stretch first overtone of amides or cellulose), and 1670 nm (first overtones of C–H stretching and N–H bonds of flavones and proteins)^43–45^.

**Figure 5:**
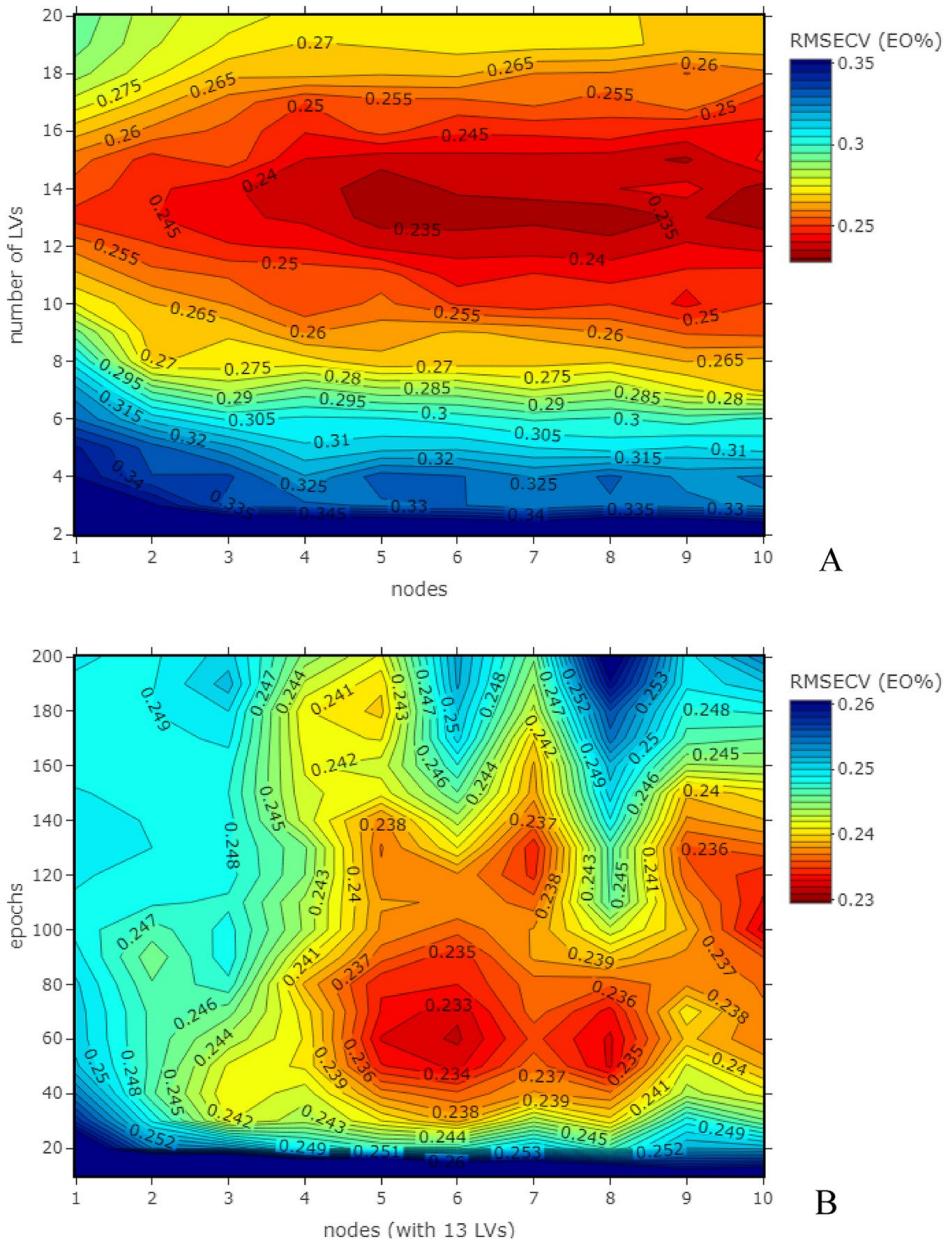
(**A**) RMSECV of the MLP models (at optimized training epochs) with increasing numbers of PLS LVs from MSC preprocessed data; (**B**) RMSECV of the MLP models based on the first 13 PLS LVs from MSC preprocessed data with varying training epochs and hidden layer nodes.

The optimal number of LVs for PLS regression of MSC preprocessed spectra was 13, as can be seen from the RMSECV values in Fig. 5A, and is shown in Table 2. When applying PLS- MLP (for mechanism see Fig. S1) a minimal in RMSECV was obtained with 13 LVs and 6 nodes (Fig. 5A). The best cross-validation was achieved when training the PLS-MLP model for 60 epochs with a RMSECV of 0.232 (R^2^ 0.844, RPD 2.53) (Fig. 5B). The associated performance indicators of prediction (RMSEP and RPDp) for this and the other models are shown in Table 2.

This is the first reported study on predicting the EO content of mint samples with hyperspectral imaging. As far as the authors know, the only other study to measure the EO concentration in a solid food product through hyperspectral imaging was done by^16^. They predicted EO in Sichuan peppers, with an EO concentration between 2.8 and 9 mL/100 g dry matter. That study worked in a region between 380 and 1040 nm, mostly in the visibly spectrum and the near end of the NIR. Contrary to this study, Ke et al.^16^ only observed improved EO prediction due to variable selection (with competitive adaptive reweighted sampling) for regression with Extreme Learning Machine, but not for SVM where usage of the full spectral information yielded better results. Slightly higher RPDs were achieved by Ke et al.^16^ than in the current study, even with PLS and SVM models while using the whole spectrum (no variable selection) and no preprocessing besides normalization of raw data (RPDs 2.8 to 3.0). Therefore, the better predictions in that study are presumably not due to different chemometric analyses. Potential reasons for slightly higher RPDs could be: (i) the spectral range of 380–1040 nm provides more useful information?, or (ii) differences in the plant matrices and EO compositions makes it hard to compare efficiency of these 2 studies.

Nonetheless, the obtained prediction in the current study (RPDp of 2.84) is good. Getting information about EO in a solid food/plant product has more interferences than when the EO is extracted in liquid form or when the model system is less complex with a smaller collection of biomolecules to influence spectral readings. For example, Ke et al.^16^ used NIR spectroscopy (1100 to 2500 nm) to quantify the monoterpene citral in spray dried, dextrin/lecithin encapsulated microparticles and reached RPD values of 2.1 (with PCR) to 4.5 (with MLP) dependent on the model type, which expresses a decent to excellent prediction in this relatively simple (few different compounds) system. Another possible complication in determining EO concentrations in a plant matrix might be that it is in essence a determination of a “group of compounds”. Determining the EO concentration is determining the sum of the quantities of various compounds. In spearmint, the EO is composed of mainly carvone and limonene, but also a number of other compounds and the exact relative abundance of the compounds may differ to some degree among different spearmint crops^8,9^. Even though it makes sense from a practical/economical point of view to determine the EO concentration of the spearmint, this potential heterogeneity of EO compounds among crops is not considered in these models. As an example, Amodio et al.^46^ determined, in fennel (*Foeniculum vulgare* Mill.) heads, the antioxidant activity (2,2-diphenyl-1-picrylhydrazyl, or DPPH method), which expresses the activity of multiple compounds within the food matrix. Antioxidant activity was then predicted, based on HSI in the Vis–NIR range (400 to 1000 nm) and the NIR range (900 to 1700 nm) and the best prediction was achieved with Vis–NIR, SNV preprocessing and PLS regression (no other regression tools were explored). This ultimately yielded an RPD of 2.14, which is at best useful for approximate predictions.

### Comparison of point measurements with surface scanning of spearmint samples in function of EO concentration prediction

**Figure 6:**
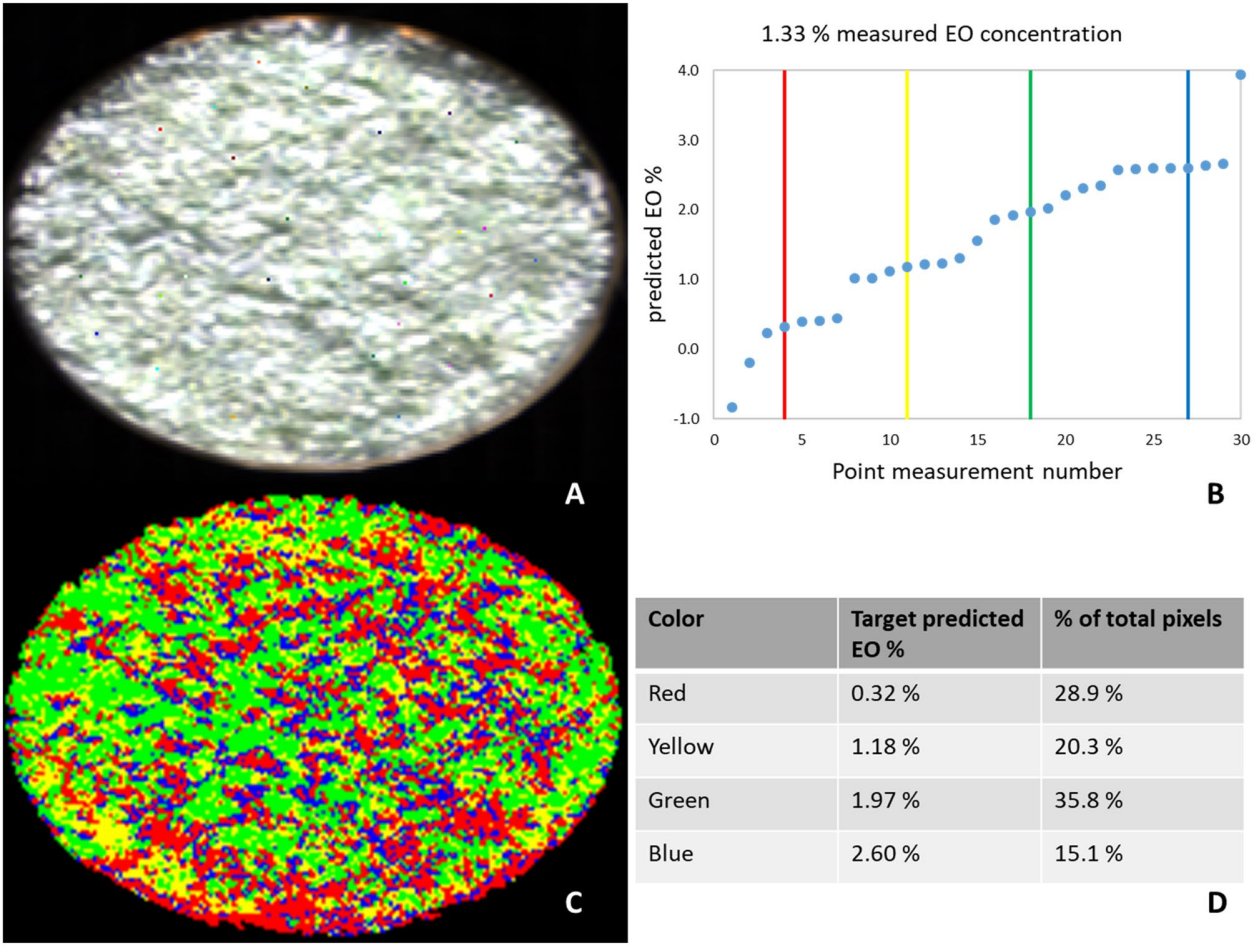
(**A**) Spearmint image (1.33% EO) with 30 selected point (pixel) measurements, shown as colored pixels. (**B**) Predicted EO % of the 30 selected points and the 4 selected target NIR spectra and associated predicted EO % represented as vertical lines in red, yellow, green and blue. (**C**) Image of the classification of all pixels into 1 of 4 categories (shown as differently colored pixels) based on resemblance of NIR spectrum at each pixel to the target spectra. (**D**) Information regarding the predicted EO % of the different color groups and the total percentage of pixels associated with each group.

The EO % predictions based on collected spectra from the 30 random point measurements are shown in Table 3. Only for the sample with 1.33% EO the mean of the point measurements prediction was not significantly different from the measured value. For the other 2 samples (0.25% and 2.45%) the mean was significantly different though. For all 3 samples the variation in predicted values was high (around 1% standard deviation). This is illustrated in Fig. S3. This large variance in prediction of EO % makes a point measurement unfeasible, even in the case where 30 points are being measured. A scanning method on the other hand where the spectra from a surface of 50 by 50 pixels (2.0 × 2.0 cm) are recorded and averaged, gave predictions with considerably lower variance and predictions closer to the measured values. To visualize the heterogeneity of the spearmint samples, a classification was done by assigning each pixel into 1 of 4 groups, based on target spectra (of 4 selected point measurements) as shown for a spearmint sample with a measured EO % of 1.33 (Fig. 6). For the samples with 0.25 EO % and 2.45 EO %, the information can be found in Figs. S4 and S5. Of the 30 random pixels (Fig. 6A), 4 were selected to serve as target NIR spectra (vertical colored lines in Fig. 6B). Selection was done to have a coverage of the different possible spectra and associated predicted EO % concentrations (Fig. 6B). From Fig. 6C,D it can be further observed that a considerable variance in NIR spectral values and predicted EO % occurred among different spatial coordinates.

**Table 3:** EO predictions of 3 spearmint samples with 0.25%, 1.33% and 2.45% EO respectively, predicted based on spectral data collected by Scan (50 × 50) pixels and by point picking of 30 random pixels.

EO %	Scan (50 × 50 pixels)		Point (30 random pixels)	
	Mean + SD	Wilcoxon signed rank (p-value)	Mean + SD	Wilcoxon signed rank (p value)
0.25	0.43 ± 0.10	0.11	0.99 ± 1.00	4 × 10^–4^
1.33	1.30 ± 0.18	1.00	1.57 ± 1.06	0.20
2.45	2.49 ± 0.19	0.79	1.79 ± 0.94	5 × 10^–4^

## Conclusions

Knowledge about EO yields is valuable, practical information, especially when obtained in a rapid, nondestructive manner. Noninvasive NIR-HSI was used to predict the EO concentration in dried spearmint and this with a good (RPD 2.84) prediction quality. Proper preprocessing (MSC or SNV) and adequate spectral variable selection, with PLS as the best technique for dimension reduction, improved the prediction quality. MLP was the better prediction tool, compared to SVM, PLS or PCR regression. This study shows that averaging the spectra of an area of HSI image pixels (50 × 50) can provide good spectral information from a heterogeneous sample with rough, uneven surface such as dried spearmint leaves. This can be done in 1 scan and without extensive handling of the sample. Predicting EO concentration based on a number of point measurements resulted in a larger variance in spectral values (and as such larger variance in EO concentration) and a less reliable estimate of the EO concentration. Looking ahead, future research should focus on (i) whether VIS-HSI might produce more useful spectral data to predict the EO concentration than NIR-HSI; (ii) whether EO concentration can rapidly be predicted with good to excellent accuracy in other relevant, commercial crops; (iii) and whether single compounds, such as carvone and limonene in spearmint but also other major EO components of importance in other crops, can be predicted nondestructively with HSI.

## Supplementary Information

Supplementary Information 1.

Supplementary Figures.

## Data Availability

The raw spectral data of mint samples and corresponding essential oil data are available in supplementary information (Spearmint_HSIspectra.xlsx).
