# Author Correction: Prediction of essential oil content in spearmint (*Mentha spicata*) via near-infrared hyperspectral imaging and chemometrics

**DOI:** 10.1038/s41598-023-36583-6

**Published:** 2023-06-09

**Authors:** Sam Van Haute, Amin Nikkhah, Derick Malavi, Sajad Kiani

**Affiliations:** 1 Department of Food Technology, Safety and Health, Faculty of Bioscience Engineering, Ghent University, Coupure Links 653, 9000 Ghent, Belgium; 2 Department of Molecular Biotechnology, Environmental Technology, and Food Technology, Ghent University Global Campus, 119, Songdomunhwa-Ro, Yeonsu-Gu, Incheon, 21985 South Korea; 3 Friedman School of Nutrition Science and Policy, Tufts University, Boston, MA USA; 4 Biosystems Engineering Department, Sari Agricultural Sciences and Natural Resources University, Sari, Iran

Correction to: *Scientific Reports* 10.1038/s41598-023-31517-8, published online 14 March 2023

The Acknowledgments section in the original version of this Article was incomplete.

“The authors gratefully acknowledge the financial support provided by Ghent University Global Campus.”

now reads:

“The authors gratefully acknowledge the financial support provided by Ghent University Global Campus and Sari Agricultural Sciences and Natural Resources University (grant number: 02-1400-02).”

The original Article has been corrected.

