# Supplementary material for: Prediction of essential oil content in spearmint (Mentha spicata) via near-infrared hyperspectral imaging and chemometrics
Source: Sci Rep. 2023 Mar 14;13:4261. doi: 10.1038/s41598-023-31517-8 (PMC10014940; doi:10.1038/s41598-023-31517-8)
Supplement: Supplementary file 2 — Supplementary Figures. [file 41598_2023_31517_MOESM2_ESM.docx]

**SUPPLEMENTARY INFORMATION**

**Coupling LASSO, PCA, PLS to MLP or SVM**

In order to use the variables from LASSO, PCA or PLS as inputs for further regression tools, a test fold needs to be kept on the side. In other words the test partition should not influence i) building the LVs, PCs or selecting the LASSO wavelengths, and ii) building the model, and only be applied as testing set.


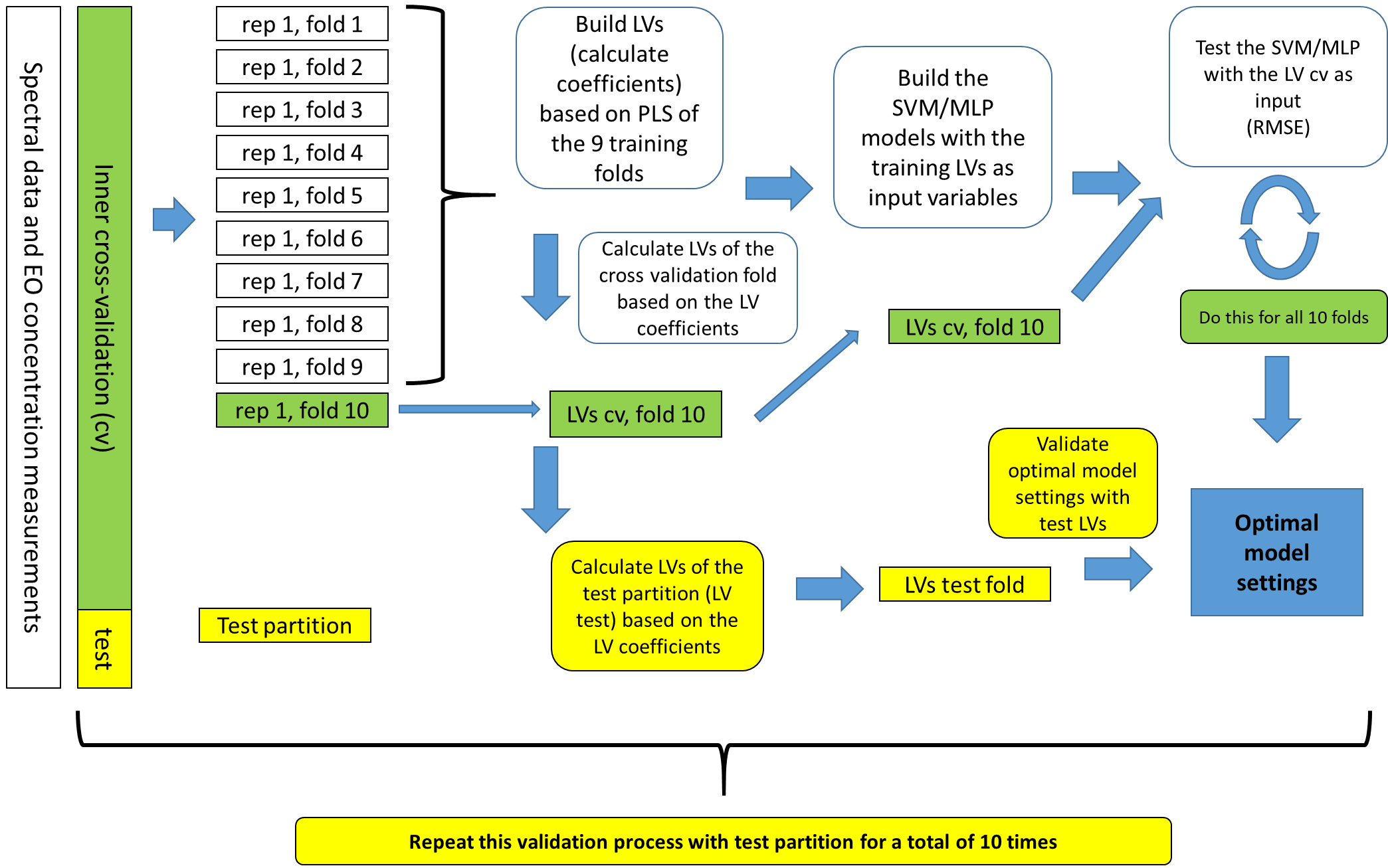


**Figure S1. Overview of coupling PLS to SVM or MLP regression tools and how to perform nested 10-fold cross-validation in those cases**

**LASSO regressionFigure S2A. Percentage inclusion of spectral variables in LASSO regression of unprocessed spectra**

**Figure S2B. Percentage inclusion of spectral variables in LASSO regression of MSC preprocessed spectra**

**Figure S2C. Percentage inclusion of spectral variables in LASSO regression of SNV preprocessed spectra**

**Figure S3. Distribution of predicted EO concentrations of point (1 pixel) measurements (n=30) of 3 spearmint samples with 0.25 %, 1.33 % and 2.45 % EO**

**
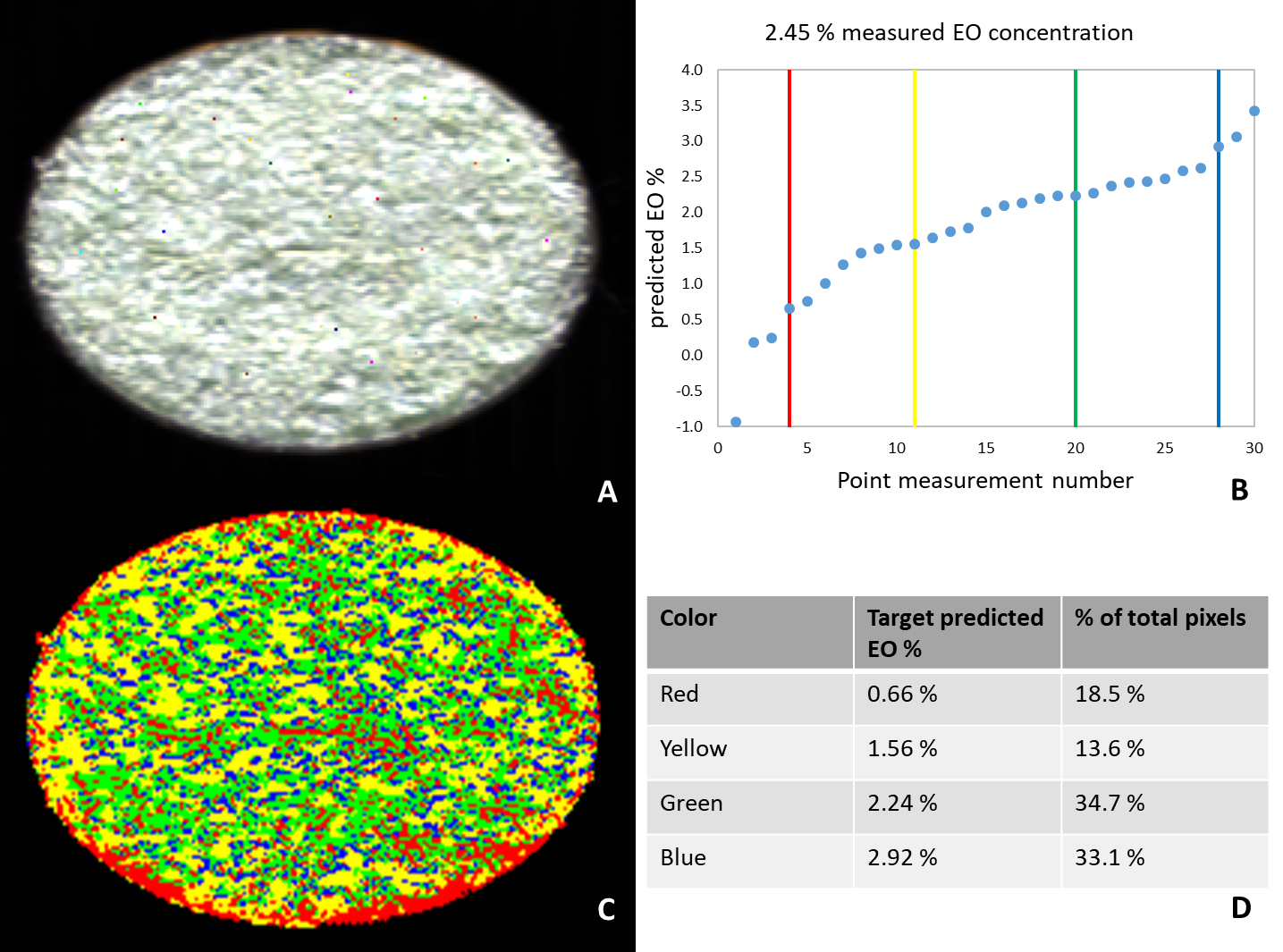
**

**Figure S4.** **Spearmint image (2.45 % EO) with 30 selected point (pixel) measurements. B. Predicted EO % of the 30 selected points and the selected target NIR spectra and associated predicted EO % represented as vertical lines in red, yellow, green and blue. C. Image of the classification of all pixels into 1 of 4 categories (shown as differently colored pixels) based on resemblance of NIR spectrum at each pixel to the target spectra. D. Information regarding the predicted EO % of the different color groups and the total percentage of pixels associated with each group.**

**
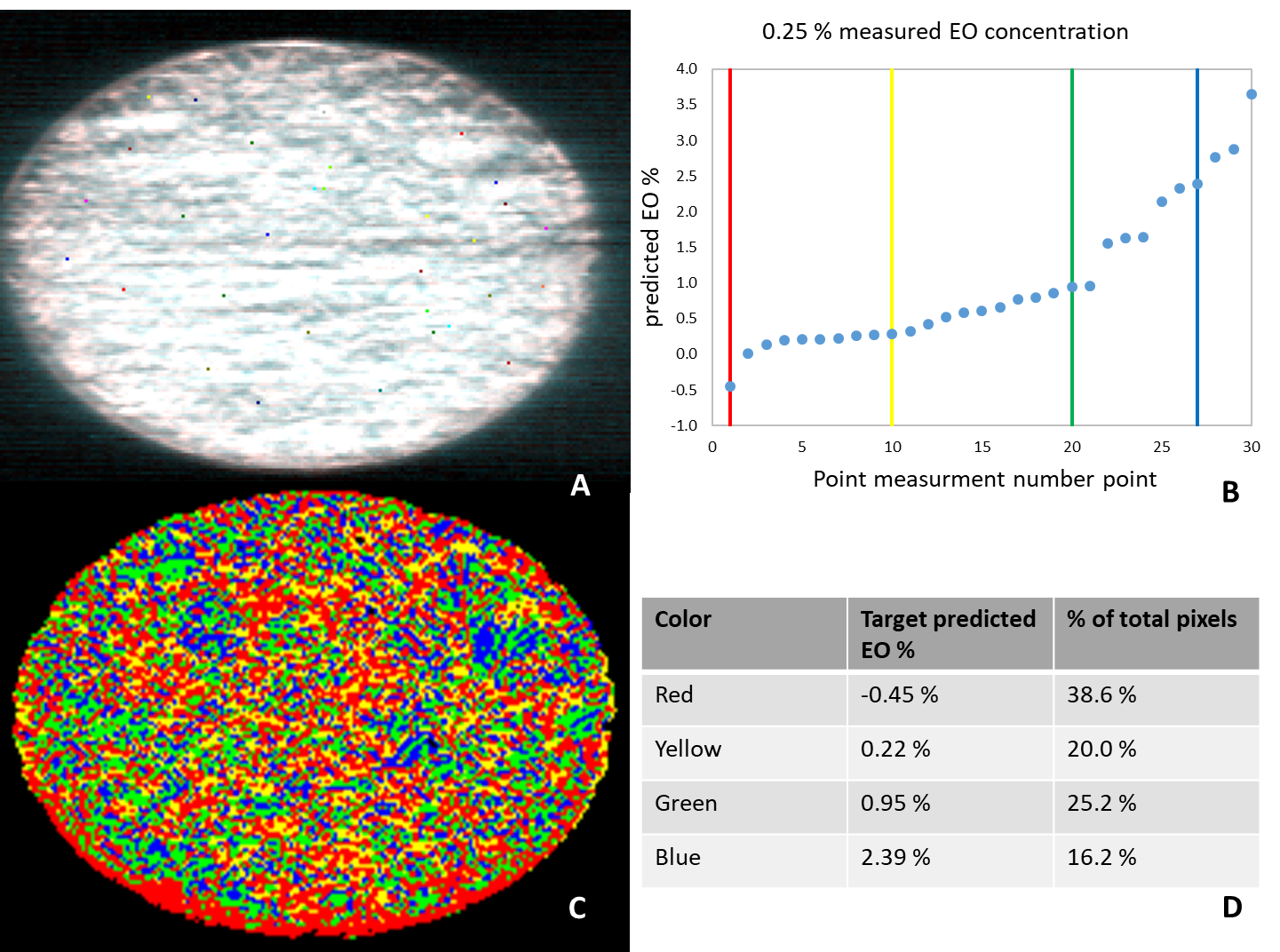
**

**Figure S5.** **Spearmint image (0.25 % EO) with 30 selected point (pixel) measurements. B. Predicted EO % of the 30 selected points and the selected target NIR spectra and associated predicted EO % represented as vertical lines in red, yellow, green and blue. C. Image of the classification of all pixels into 1 of 4 categories (shown as differently colored pixels) based on resemblance of NIR spectrum at each pixel to the target spectra. D. Information regarding the predicted EO % of the different color groups and the total percentage of pixels associated with each group.**

***Calculation of predicted EO % of the entire image based on the 4 groups.***

**Predicted % EO = Red conc. x (% total pixels)/100 + Yellow conc. x (% total pixels)/100+ Green conc. x (% total pixels)/100+ Blue conc. x (% total pixels)/100**

**0.25 % 🡪 0.51 %**

**2.45 % 🡪 2.08 %**

**1.33 % 🡪 1.43 %**
